# The impact of weather conditions on avian breeding performance: insights from a long-term study

**DOI:** 10.1186/s12983-025-00569-z

**Published:** 2025-08-25

**Authors:** Aneta Arct, Rafał Martyka, Krzysztof Miler, Karolina Skorb, Lars Gustafsson, Szymon M. Drobniak

**Affiliations:** 1 Institute of Environmental Sciences, Faculty of Biology, Jagiellonian University, Gronostajowa 7, 30-387 Kraków, Poland; 2 Animal Ecology, Department of Animal Ecology/Ecology and Genetics, Evolutionary Biology Centre, Uppsala University, Norbyvägen 18 D, 752 36 Uppsala, Sweden; 3 Institute of Nature Conservation, Polish Academy of Sciences, Mickiewicza 33, 31-120 Kraków, Poland; 4 Institute of Systematics and Evolution of Animals, Polish Academy of Sciences, ul. Sławkowska 17, 31-016 Kraków, Poland; 5 Doctoral School of Natural and Agricultural Sciences, W. Szafer Institute of Botany PAS, Lubicz 46, 31-512 Kraków, Poland; 6 Evolution and Ecology Research Centre, School of Biological, Environmental and Earth Sciences, University of New South Wales, Kensington, 2052 Australia

**Keywords:** Climate change, Thermal conditions, Reproductive success, Passerine birds, Collared flycatcher (*Ficedula albicollis*)

## Abstract

Understanding how weather conditions during early development influence reproductive success is essential for predicting avian responses to climate change. We used a 40-year dataset from a nest-box population of collared flycatchers (*Ficedula albicollis*) to examine how temperature and precipitation during the incubation and nestling periods affected three key components of reproductive performance: hatchling number, fledgling production, and local offspring recruitment. We found that higher ambient temperatures during the nestling period were associated with a decreased probability of brood failure and a higher number of recruits. In contrast, a higher sum of precipitation during the nestling stage was associated with an increased likelihood of brood failure. Interestingly, weather conditions during incubation had no statistically detectable influence on reproductive performance. Moreover, our results indicated that the within-season variation in climatic conditions, rather than the between-season variation, was primarily associated with reproductive outcomes. These findings underscore the importance of considering both overall climate conditions across seasons and short-term weather parameters within breeding seasons when evaluating reproductive success and predicting the ecological consequences of climate change.

**Supplementary Information:**

The online version contains supplementary material available at 10.1186/s12983-025-00569-z.

## Introduction

Climate profoundly impacts dynamics and long-term trends in wildlife populations [5, 30]. Dramatic population declines are observed in many avian groups [47], and difficulties in coping with climate change may largely contribute to the observed declines. Populations may persist or perish depending on how individual organisms respond to environmental changes. Predicting how species will respond to anthropogenic climate change is one of the most pressing challenges in contemporary ecology and conservation biology, because accurate forecasts are essential for guiding protection and management actions [27, 59].

Birds are good anthropogenic disturbance indicators and valuable for evaluating climate change impacts [16]. For avian species whose reproductive success depends on resource availability shaped by microclimatic conditions, environmental change poses significant challenges to their overall survival. Unexpected weather might generate mismatches between offspring demands and food availability [57], as observed in many species (e.g., [18]). Although numerous species attempt to respond to climate change through shifts in phenology (e.g., [25, 37], see [44] for a meta-analysis), such adjustments are not always possible. For example, pied flycatchers exhibit limited phenological plasticity [7]. Moreover, once clutch initiation has occurred, parents generally have little behavioural or physiological flexibility to buffer their offspring against subsequent adverse weather conditions. Weather conditions can have varying impacts at different life stages, however, early life stages appear to be the most sensitive window to any environmental conditions. Growing evidence suggests that early life stages, along with their crucial role in developmental plasticity, are particularly important in shaping the lifetime fitness of individuals [26, 36]. In particular, the temperature during pre- and post-hatching development has the potential to affect the physiology and condition of developing precursor tissues of a growing embryo and nestling, and such alterations may, in turn, affect offspring condition and survival [3, 8, 26, 38]. Although many studies have explored how weather conditions during development affect offspring performance, our understanding of the influence of early life conditions on offspring performance remains limited and context-dependent [12, 13, 34, 41, 54, 61].

Long-term studies focusing on reproduction and climate change are essential for understanding how environmental shifts impact avian reproductive patterns [45]. Here, we used a 40-year dataset from a wild population of collared flycatchers (*Ficedula albicollis*) breeding on the Swedish island of Gotland to investigate how ambient temperature and precipitation during the incubation and nestling periods influence key reproductive components, including the number of hatchlings, fledglings, and locally recruiting offspring. Our research question is particularly relevant because the study population has experienced a marked increase in ambient temperature during the breeding season across the study period (Table SM1, Fig. SM1). Although we observed no change in average rainfall over the years (Table SM1, Fig. SM2), precipitation can have a strong influence on food availability and other ecological conditions that are important for reproductive success [55]. The collared flycatchers feed nestlings almost exclusively with caterpillars and flying arthropods, the abundance and activity of these prey decline in prolonged rain [50] and during heat or cold extremes that suppress larval foraging [52]. Adverse weather may also increase nestling thermoregulatory costs and can trigger brood reduction or complete failure [42]. Additionally, prolonged precipitation increases nest humidity, which can promote the growth of pathogens and chill nestlings, thereby compromising their survival [42]. Excessive rainfall may also reduce food availability, further increasing thermal and disease-related stress in nestlings. Taken together, these effects suggest that increased precipitation during the incubation and nestling periods is likely to negatively affect reproductive success.

Similarly, average temperatures that deviate from optimal ranges during the nestling period may impair developmental trajectories and reduce post-fledging survival [10]. While both cold and heat extremes can negatively affect reproductive success, our predictions focus on the impact of higher temperatures. This emphasis reflects observed climatic trends in our study population, where ambient temperatures during the breeding season have increased over time (Table SM1, Fig. SM1). Therefore, we predict that elevated temperature during incubation and nestling periods will reduce the number of hatched nestlings, fledgling production, and local offspring recruitment.

To better disentangle the effects of climatic variability at different temporal scales, we partitioned both temperature and precipitation into between-year (seasonal means) and within-year (deviations from the seasonal mean per brood) components. This approach allows us to test whether both between-season effects of weather conditions (interannual variation) and within-season effects of weather conditions (intraannual variation) influence reproductive parameters.

## Methods

### Study species

The collared flycatcher is a small (~ 13 g) migratory passerine bird. The birds arrive from their wintering areas in southern Africa to the breeding areas in late April to mid-May. The collared flycatcher is a single-brooded species and usually lays one clutch consisting of six eggs on average (range 4–8). However, if a breeding attempt fails before fledging and sufficient time remains in the breeding season, breeding pairs may initiate a replacement brood [20]. At the beginning of May, the first eggs are laid, and incubation begins, a task undertaken solely by females. Nestlings hatch after approximately 13 days of incubation [1, 28], remain in the nests for an additional 14–16 days, and are fed by both parents. They reach the maximum body mass at the age of 10–11 days and lose some mass before fledging. Then, fledglings stay close to the nest for another two weeks and are still fed by their parents. Monitoring of the population biology of the collared flycatcher has been ongoing at Burgsvik (Gotland, Sweden; 57° 03′  N, 18° 17′  E) since 1980. The analyses presented here are based on a 40-year subset of data collected between 1980 and 2019. The collared flycatcher population on Gotland is isolated from the main species range, and so recapture and return rates of individuals are high [20]. The high occupancy rates of nest boxes by this species facilitate detailed individual-based monitoring and experimental approaches in natural settings [20]. Based on long-term monitoring, the average recruitment rate in the Gotland population of collared flycatchers is estimated to be approximately 15% [23], unpublished data).

### General procedures

In the studied nest box population of the collared flycatcher, breeding birds were monitored over the whole season to gather data including laying date, clutch size, brood size, and the number of fledglings and recruits [35]. Females were trapped at the nest during incubation (May–June), after catching, each bird was banded with a metal band and aged as 1 year old or older [53]. Age was determined based on plumage characteristics and ringing history. Birds ringed as nestlings in previous years were assigned exact ages. For others, we classified them as “young” if they were in their second calendar year (first breeding season), and “old” if they were older than two years. All breeding attempts were carefully monitored until the chicks fledged. On day 12 after hatching, nestlings were measured and banded with a metal band. In the study, we used the longitudinal dataset, containing all the records on annual female reproductive performance (i.e., the number of hatched nestlings and fledglings reared to independence and the number of recruits from each breeding attempt). However, for recruitment rate analyses, we used data only from the years 1980 to 2016 to ensure completeness and comparability across cohorts, allowing sufficient time for individuals to return and be detected as local recruits. We excluded from the dataset all breeding events that were involved in any experimental manipulations. Sample sizes differ among analyses because some nests lacked complete field data (e.g., missing records on hatching or fledging success).

### Climatic factors

We chose average daily temperature and daily sums of precipitation as they are commonly used metrics in ecological studies, providing a balanced representation of overall weather conditions. Daily temperature records and daily sums of precipitation were obtained from the meteorological station at Hoburgen (56.92° N, 18.15° E; approximately 10 km from the main study areas). The data were accessed via the website of the Swedish Meteorological and Hydrological Institute (SMHI), which provides pre-calculated daily mean temperatures and precipitation sums (details on the calculation methods used by SMHI can be found on their website http://opendata-download-metobs.smhi.se/explore/?parameter=3).

Although hatching dates were available for a large number of nests, this information was missing for a substantial proportion of records across the 40-year dataset. In earlier years of the study, hatch dates were more frequently missing due to less intensive monitoring, whereas in later years, improved field protocols led to more complete data collection. To avoid excluding a large number of observations and introducing temporal bias, we adopted a standardised approach by assigning a fixed incubation and nestling period to all nests. For the incubation period, we calculated the average daily temperature and the daily sums of precipitation over 13 days, starting from the day the last egg was laid. We chose the 13 days because previous work by Arct et al. [1] showed that the mean incubation duration in this collared flycatcher population was 13.2 days (range 12–17 days).

Furthermore, fledging dates were inconsistently recorded, preventing us from using brood-specific timing in the analysis of weather effects. To extract weather conditions relevant to the nestling stage, we used a 15-day window beginning on the final day of the incubation period. This period was chosen as a standardised approximation of the nestling phase across the dataset. While temperature logger data from a small subset of nests (N = 10) indicate that fledging typically occurs around day 16.2 (range: 14–19 days; J. Sudyka, unpublished data), the majority of nests lack precise fledging dates. Given the natural variability in fledging timing and limitations in accurately determining fledging age at the population level, we adopted a 15-day window.

### Statistical analysis

We used generalized linear mixed models (GLMMs) to examine how weather conditions during the incubation and nestling periods affect three components of reproductive success: the number of hatched nestlings (hatching success), the number of fledglings (fledgling production), and the number of locally recruiting offspring (local offspring recruitment). For each reproductive component, we constructed separate models based on relevant data subsets: the hatchling number model included all clutches, the fledging production model included only broods where at least one egg hatched, and the local recruitment rate model included only broods that produced at least one fledgling. This approach avoids compounding zero values across sequential reproductive stages.

Hatchling number was modelled using a GLMM with a Gaussian error distribution and identity-link function. Due to an excess of zeros in the fledgling count data, we applied a zero-inflated GLMM with Poisson error variance and log-link function for a conditional component and binomial error variance and logit-link function for a zero-inflated component to analyse offspring production. Recruitment data, in contrast, did not show severe zero inflation and were analysed using a GLMM with Poisson error distribution and log-link function.

In all models, we used the averaged ambient temperature and the sum of precipitation experienced during the incubation and/or nestling period as terms describing climatic conditions. Both terms were calculated as within- (mean value calculated per brood subtracted from overall mean value in the season, i.e., within-season mean centering) and between-year effect (overall mean value calculated per season) to separate the impact of those two different sources of variation on reproductive parameters. However, it is important to note that high synchrony in laying dates within the study population may limit the extent of within-season variation in weather conditions experienced by different broods.

Moreover, we also included female age (categorical predictor: young versus old birds), female body condition (calculated as scaled mass index; see [40]), laying date, and clutch size in all models. Female age and body condition are factors that are expected to influence reproductive investment and sensitivity to environmental conditions in our population (e.g., [35, 48]). In turn, laying date was included because timing of breeding is known to affect reproductive success in collared flycatchers, with early breeders typically experiencing more favourable environmental conditions and higher offspring survival (e.g., [11, 60]). Clutch size accounted for maternal investment in the number of produced offspring.

To control for repeated measurements and spatial structure, we included female identity, study plot, and year as random factors in all models. All continuous predictors were standardised using z-transformation (mean = 0, SD = 1). Models were fitted using the glmmTMB package (ver. 1.1.9) in R (ver. 4.3.3; [9, 43]). We assessed model fit and assumptions using diagnostics offered by the DHARMa (ver. 0.4.6; [24]) and the performance (ver. 0.12.2 [33],) packages. For each model, we calculated marginal and conditional R^2^ or pseudo-R^2^, as appropriate, using the MuMIn package (ver. 1.48.4; [6]). Potential multicollinearity was assessed based on both variance inflation factors (VIFs) calculated in the performance package and correlation matrices, all VIF values and correlation coefficients did not exceed 3.80 and 0.65, respectively, indicating acceptable levels for maintaining proper model performance [15], full details and the corresponding table are provided in the Table SM 2,3).

## Results

### Hatchling number

Our analyses indicate that the number of hatched nestlings depended on female age, female body condition, and clutch size (Table 1). Young, inexperienced females had fewer hatchlings than old ones, whereas females in poorer body condition produced more hatchlings than better-conditioned females (Table 1). The number of hatchlings was positively associated with clutch size.

**Table 1:** Effects of female traits and climatic variables on the number of hatchlings

Sources of variation	Estimate	SE	z	*P*
*(N* = *8895* *broods* *of 6457 females)*				
Intercept	**5.54**	**0.03**	**204.93**	** < 0.001**
Female age: young	**− 0.04**	**0.02**	**− 2.06**	**0.040**
Female body condition	**− 0.03**	**0.01**	**− 3.08**	**0.002**
Within-year temperature: incubation	0.00	0.01	0.39	0.70
Between-year temperature: incubation	0.04	0.02	1.72	0.085
Within-year precipitation: incubation	0.01	0.01	0.91	0.36
Between-year precipitation: incubation	− 0.01	0.02	− 0.32	0.75
Laying date	− 0.03	0.02	− 1.76	0.079
Clutch size	**0.40**	**0.01**	**31.23**	** < 0.001**
Female ID _random_	1.25			
Plot ID _random_	0.03			
Year of study _random_	0.11			
R^2^_marginal/conditional_	0.09/0.89			

Output of generalized linear mixed model with Gaussian error distribution and the identity-link function testing how female age (as a categorical predictor), female body condition, the within- and between-year effects of ambient temperature and sum of precipitation experienced during the incubation period, laying date, and clutch size (all as continuous predictors), affect the number of hatchlings. All continuous explanatory terms were standardized. The female identity, study plot identity, and year of study were included as random factors. Significant terms *P* < 0.05 are in bold

### Fledgling production

**Fig. 1:**
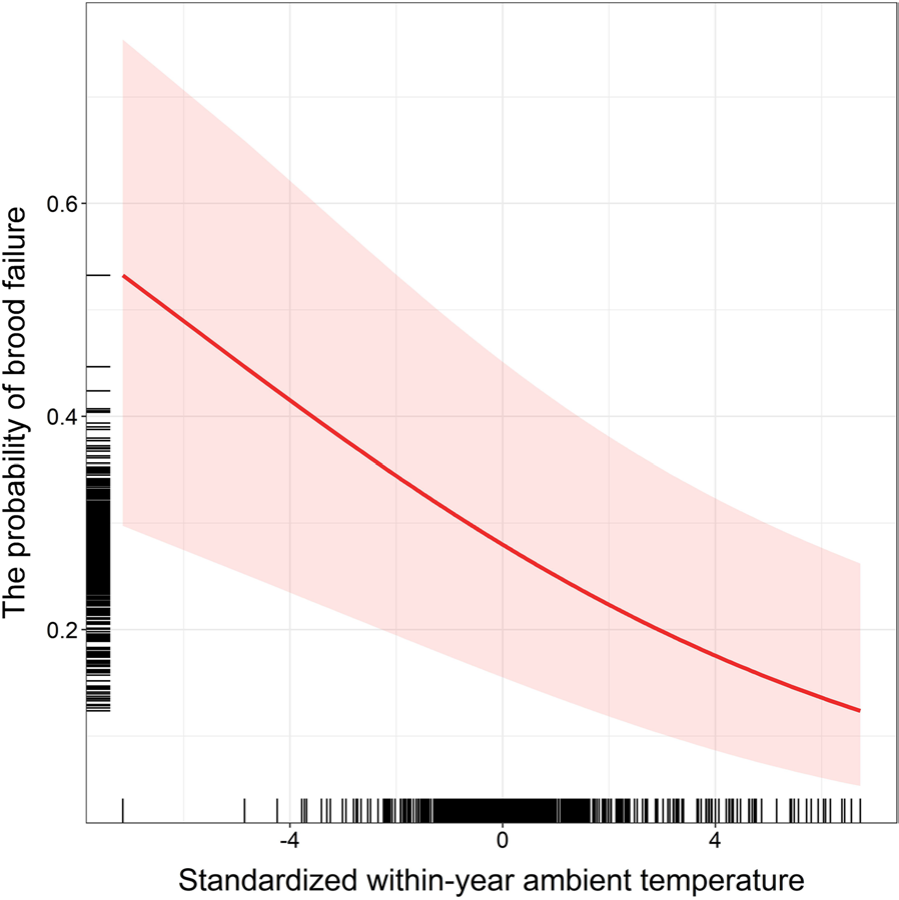
The probability of brood failure in relation to the within-year ambient temperature during the nestling period. A predicted trend accompanied by 95% confidence intervals from the model (in red) and distribution of data points along both axes (black rugs) are shown

**Fig. 2:**
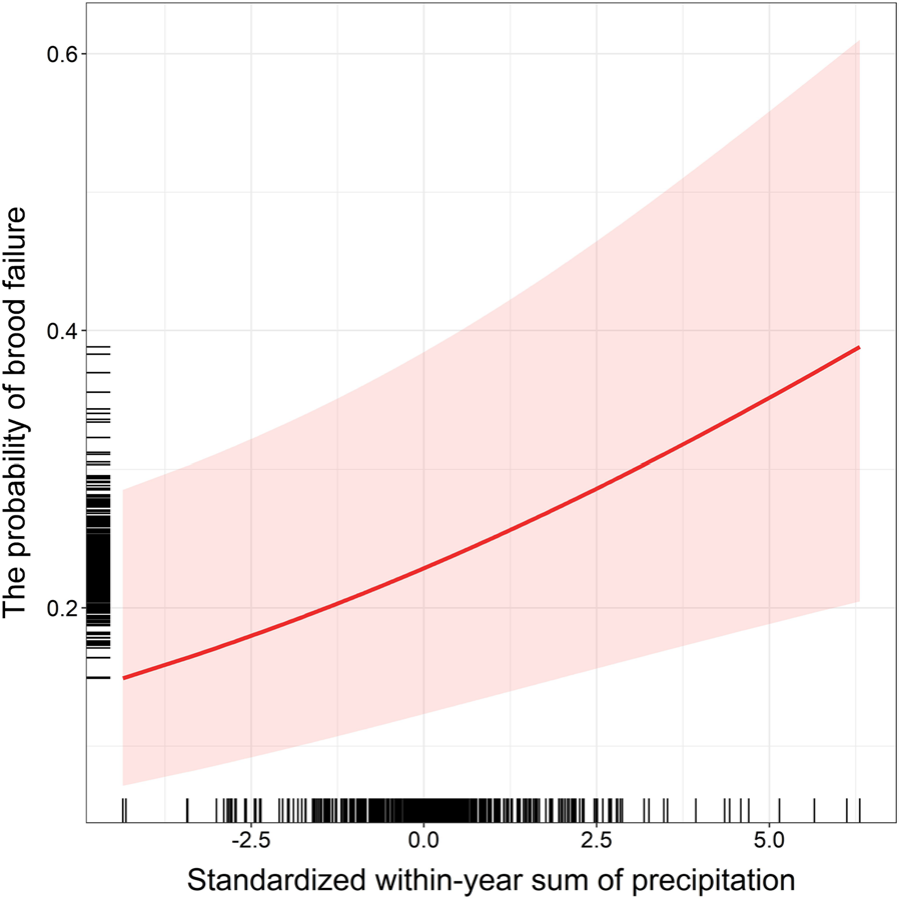
The probability of brood failure in relation to the within-year sum of precipitation during the nestling period. A predicted trend accompanied by 95% confidence intervals from the model (in red) and distribution of data points (black rugs) are shown

The number of produced fledglings was affected by laying date and clutch size. In contrast, the probability of brood failure (i.e., producing no fledglings) depended on within-year ambient temperature and the sum of precipitation, as well as laying date and clutch size (Table 2). Specifically, the fledgling number was positively associated with clutch size and negatively related to laying date, indicating that larger and earlier clutches produced more fledglings (Table 2). In turn, the probability of brood failure decreased as within-season ambient temperature increased during the nestling period (Table 2, Fig. 1). In contrast, the higher within-season sum of precipitation during the nestling period was associated with an increased probability of brood failure (Table 2, Fig. 2). Laying date was positively related to brood failure probability, indicating that later-breeding females were more likely to fledge no offspring (Table 2). Clutch size was also positively associated with the probability of brood failure, implying that females laying larger clutches had a higher risk of reproductive failure (Table 2).

**Table 2:** Effects of female traits and climatic variables on the number of fledglings

Sources of variation	Estimate	SE	z	P
*Conditional model (N* = *7315 broods of 5513 females)*				
Intercept	**1.52**	**0.02**	**69.11**	** < 0.001**
Female age	− 0.01	0.01	− 0.83	0.41
Female body condition	0.00	0.01	− 0.13	0.90
Within-year temperature: incubation	0.00	0.01	0.43	0.67
Between-year temperature: incubation	0.01	0.02	0.47	0.64
Within-year precipitation: incubation	0.00	0.01	0.16	0.87
Between-year precipitation: incubation	0.03	0.02	1.71	0.088
Within-year temperature: nestlings	0.02	0.01	1.44	0.15
Between-year temperature: nestlings	− 0.02	0.02	− 0.86	0.39
Within-year precipitation: nestlings	− 0.01	0.01	− 1.79	0.073
Between-year precipitation: nestlings	− 0.04	0.02	− 1.69	0.092
Laying date	− **0.07**	**0.02**	− **4.07**	** < 0.001**
Clutch size	**0.09**	**0.01**	**12.38**	** < 0.001**
Female ID _random_	0.00			
Plot ID _random_	0.05			
Year of study _random_	0.10			
Pseudo-R^2^_marginal/conditional_	0.08/0.13			
*Zero-inflated model (N* = *7315 broods of 5513 females)*				
Intercept	− **1.96**	**0.18**	− **11.03**	** < 0.001**
Female age	0.07	0.08	0.87	0.38
Female body condition	0.04	0.04	1.16	0.24
Within-year temperature: incubation	− 0.07	0.05	− 1.47	0.14
Between-year temperature: incubation	− 0.28	0.19	− 1.44	0.15
Within-year precipitation: incubation	− 0.02	0.04	− 0.53	0.60
Between-year precipitation: incubation	− 0.19	0.14	− 1.32	0.19
Within-year temperature: nestlings	− **0.15**	**0.04**	− **3.45**	** < 0.001**
Between-year temperature: nestlings	0.25	0.20	1.22	0.22
Within-year precipitation: nestlings	**0.12**	**0.04**	**2.94**	**0.003**
Between-year precipitation: nestlings	0.31	0.18	1.78	0.076
Laying date	**0.73**	**0.08**	**8.94**	** < 0.001**
Clutch size	**0.11**	**0.04**	**2.86**	**0.004**
Female ID _random_	0.00			
Plot ID _random_	0.47			
Year of study _random_	0.87			

Output of the zero-inflated generalized linear mixed model with Poisson error distribution and the log-link function for a conditional component and with binomial error distribution and the logit-link function for a zero-inflated component testing how female age (categorical predictor), female body condition, the within- and between-year effects of ambient temperature and sum of precipitation experienced during the incubation and nestling period, laying date, and clutch size (all as continuous predictors), affect the number of fledglings. All continuous explanatory terms were standardized. The female identity, study plot identity, and year of study were included as random factors. Significant terms *P* < 0.05 are in bold

### Local offspring recruitment

**Fig. 3:**
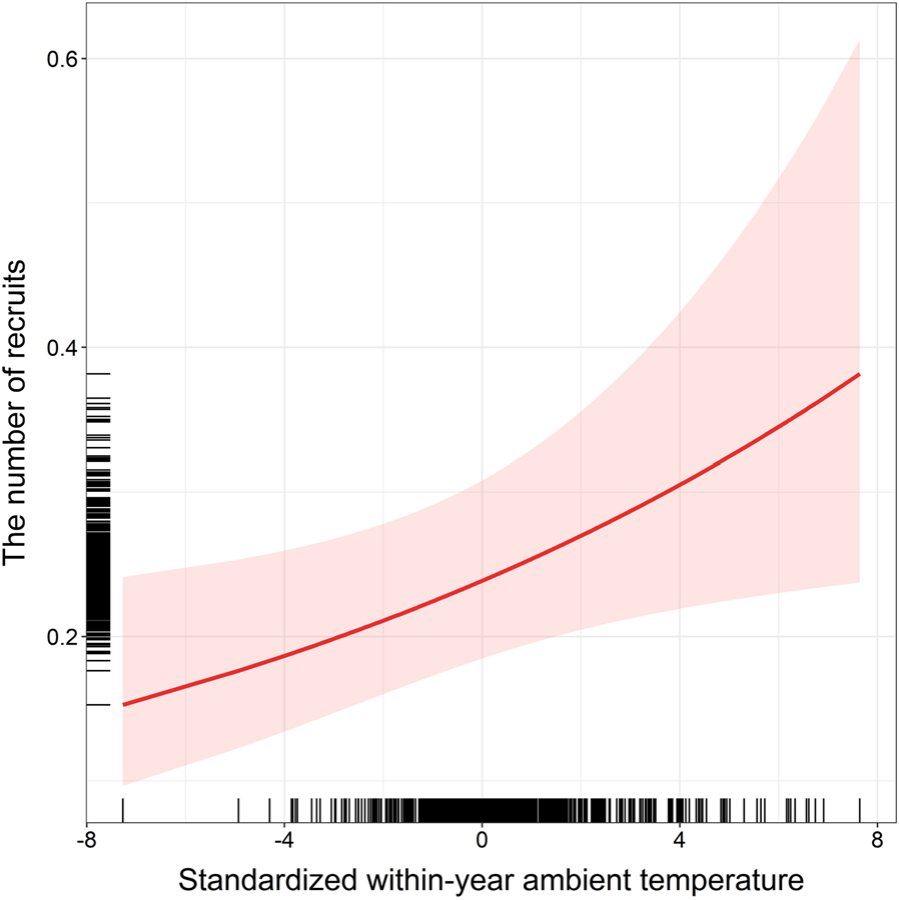
The number of recruited offspring in relation to the within-year ambient temperature during the nestling period. A predicted trend accompanied by 95% confidence intervals from the model (in red) and distribution of data points (black rugs) are shown

Offspring recruitment was affected by the within-year ambient temperature, laying date, and clutch size (Table 3). The number of recruited offspring per brood increased when nestlings experienced higher within-season ambient temperatures (Table 3, Fig. 3). Clutch size was positively associated with recruitment, indicating that larger broods had a higher likelihood of producing locally recruiting offspring (Table 3). In contrast, laying date had a negative effect on offspring recruitment, with later breeding being associated with lower recruitment (Table 3). There was also a marginally non-significant tendency for females in better condition to produce fewer recruits (Table 3).

**Table 3:** Effects of female traits and climatic variables on the number of recruits

Sources of variation	Estimate	SE	z	*P*
*Conditional model (N* = *7315 broods of 5513 females)*				
Intercept	− **1.43**	**0.13**	− **11.25**	** < 0.001**
Female age	0.03	0.04	0.73	0.47
Female body condition	− 0.03	0.02	− 1.89	0.059
Within-year temperature: incubation	0.00	0.02	− 0.13	0.90
Between-year temperature: incubation	0.01	0.10	− 0.01	0.99
Within-year precipitation: incubation	0.02	0.02	1.20	0.23
Between-year precipitation: incubation	0.11	0.07	1.51	0.13
Within-year temperature: nestlings	**0.06**	**0.03**	**2.31**	**0.021**
Between-year temperature: nestlings	0.03	0.10	0.26	0.79
Within-year precipitation: nestlings	0.00	0.02	− 0.05	0.96
Between-year precipitation: nestlings	− 0.06	0.08	− 0.75	0.45
Laying date	− **0.32**	**0.05**	− **6.76**	** < 0.001**
Clutch size	**0.04**	**0.02**	**2.13**	**0.033**
Female ID _random_	0.22			
Plot ID _random_	0.69			
Year of study _random_	0.43			
Pseudo-R^2^_marginal/conditional_	0.02/0.22			

Output of the generalized linear mixed model with Poisson error distribution and the log-link function testing how female age (categorical predictor), female body condition, the within- and between-year effects of ambient temperature and sum of precipitation experienced during the incubation and nestling period, laying date, and clutch size (all as continuous predictors), affect the number of recruits. All continuous explanatory terms were standardized. The female identity, study plot identity, and year of study were included as random factors. Significant terms *P* < 0.05 are in bold

## Discussion

Our study demonstrated that climatic conditions influence offspring production and recruitment of the migratory collared flycatcher, aligning with existing research on population ecology and climate change that shows a strong impact of climatic factors on crucial life history parameters (e.g., [12, 13, 16, 31, 32, 34, 54]). Several studies have documented that offspring production (e.g., the number of fledglings) in avian populations is linked to local weather conditions. Some studies show negative effects of high temperatures on immediate post-fledging survival [19, 46], but others report increased fledgling production during warmer and more stable breeding seasons [21, 58]. Many studies also failed to show any impacts of weather conditions on offspring production altogether (e.g., [17, 49]. Inconsistent findings may stem from relatively small sample sizes, as larger studies often show weak but biologically relevant relationships between abiotic conditions and reproductive output. In our study, although the absolute effect sizes of temperature and precipitation were generally small, their repeated, stage-specific influence across multiple components of reproductive success suggests a cumulative and biologically meaningful impact. A global meta-analysis indicates that climate change does not directly correlate with avian offspring production, but interacts with species-specific life history and ecological traits [22]. Local climatic conditions that vary seasonally and geographically can confound global trends [51]. Future research should focus on how various aspects of ecology and life history drive variation in offspring production within species or populations and across species. Our study and a recent study of Riggio et al. [45] highlight the importance of long-term monitoring to understand the impacts of climate change on reproductive performance.

We found that weather conditions during the incubation period did not significantly affect key components of reproductive output, such as the number of fledglings or locally recruited offspring. In contrast, and consistent with a recent meta-analysis by Halupka et al. [22], conditions during the nestling period had a stronger impact. Specifically, higher within-season temperatures were associated with a lower probability of complete brood failure. Conversely, lower within-season temperatures and higher total precipitation were linked to increased brood failure and reduced fledgling production. These findings suggest that unfavourable weather conditions during nestling development, particularly cooler or wetter periods within a season, can negatively affect reproductive success, likely by limiting food availability or increasing thermoregulatory costs.

As an insectivore, the collared flycatcher may be particularly affected by changing rainfall patterns, which reduce prey availability [4]. Our results confirm previous studies showing the negative effects of rainfall on the number of fledglings [2, 14, 39]. While higher temperatures can reduce reproductive failure, increased rainfall during critical developmental periods can negatively impact offspring survival [42, 50]. These findings underscore the need to consider both temperature and precipitation in understanding how climate change influences avian reproductive success.

The number of recruits reflects the individual contribution to the breeding population [56]. However, we should keep in mind that this fitness estimate does not include all offspring that survive and disperse out of the study population. Nevertheless, the decrease in the number of recruits can have more profound implications for the size and sustainability of a bird population than the number of fledglings. This suggests that recent observed population declines across the globe may reflect changes in juvenile survival [47]. Here, we used the yearly number of recruits, and we found a strong positive effect of higher ambient temperature during the nestling phase on the number of recruits in the wild population of collared flycatcher.

In a previous study of the same population, we manipulated the developmental conditions of embryos through a modification of nest box thermal microclimate, and found that offspring from the experimentally heated nests had larger body mass at fledging in comparison to the control ones, which is associated with higher future survival [3]. This suggests that the increase in ambient temperature during the breeding season may benefit the reproductive success of collared flycatcher inhabitants on Gotland Island.

While our findings suggest that higher average temperatures during the nestling period may reduce reproductive failure and increase recruitment, it is important to consider not only average values but also the variability and interactions among climatic parameters. Recent studies have highlighted that spring temperatures are becoming increasingly unpredictable due to climate change, which can lead to reduced reproductive success [49, 54]. Moreover, climate change is not only associated with rising temperatures but also with shifts in precipitation regimes. The Intergovernmental Panel on Climate Change (IPCC, [29]) projects an increase in the frequency and intensity of extreme precipitation events across many regions, including Europe. These changes may adversely affect avian reproduction by reducing food availability or increasing energetic costs during the nestling phase. Future studies should therefore aim to explicitly test for interactive effects between temperature and precipitation, as this may offer a more ecologically realistic understanding of how climate change shapes avian reproduction.

In addition to climatic variables, our results highlight the consistent influence of laying date and clutch size on reproductive performance across all stages examined. Early breeders produced significantly more fledglings and locally recruited offspring, in line with previous findings in collared flycatchers (e.g., [11, 60]). This likely reflects seasonal declines in food availability and deteriorating environmental conditions later in the breeding season. Similarly, larger clutches were associated with higher numbers of hatchlings, fledglings, and recruits.

Interestingly, our analysis revealed a positive association between clutch size and the probability of brood failure (Table 2). This pattern is counterintuitive, as larger clutches are typically associated with higher reproductive investment and, under favourable conditions, greater reproductive success. One possible explanation is that larger clutches may be more vulnerable to environmental stressors or parental limitations, especially under suboptimal weather conditions. Alternatively, some females may overestimate their capacity to rear large broods, particularly in early or unusually warm seasons, which can lead to a higher risk of complete brood loss. This result may suggest a potential trade-off between clutch size and the ability to buffer environmental unpredictability, and it merits further investigation in future studies.

These results emphasise that parental investment traits such as breeding timing and clutch size are central to reproductive performance. Moreover, in all models, we controlled for female age and body condition to account for differences in parental quality. The results revealed an unexpected pattern: females in better condition had fewer hatchlings. This result may reflect a trade-off strategy, where females in better condition allocate resources toward self-maintenance or future reproduction rather than maximising current reproductive output. It is also possible that females in poorer condition prioritise current reproduction due to lower expected future reproductive success, a pattern observed in other bird species facing uncertain survival prospects. In contrast, the negative association between age and hatchling number was consistent with previous studies, as younger females are typically less experienced (e.g., [48]).

To conclude, our results suggest that within-season weather patterns, rather than between-year variation in temperature and precipitation, are more strongly associated with reproductive outcomes. Specifically, higher within-season temperatures during the nestling period reduced brood failure, whereas increased within-season precipitation increased brood failure and reduced fledgling production. These findings highlight the sensitivity of avian reproductive output to fine-scale environmental variation. This finding underscores the importance of incorporating short-term, localised weather conditions in studies of climate impacts on avian reproduction, especially in long-term datasets where inter-annual means may mask ecologically meaningful within-season heterogeneity.

## Supplementary Information

Additional file1 (DOCX 304 KB)

## Data Availability

Our data are made publicly available via Dryad at 10.5061/dryad.w9ghx3g06. ReviewerURL: http://datadryad.org/stash/share/gE_Uxx0BZYC7U9uv2tVyhaKR6lug7cJkossLgWfI9qk.
