# Supplementary material for: The impact of weather conditions on avian breeding performance: insights from a long-term study
Source: Front Zool. 2025 Aug 25;22:23. doi: 10.1186/s12983-025-00569-z (PMC12376468; doi:10.1186/s12983-025-00569-z)
Supplement: Supplementary file 1 — Additional file1 (DOCX 304 KB) [file 12983_2025_569_MOESM1_ESM.docx]

**Supplementary material**

Table SM1. Outputs of the general linear model (GLM) testing the effects of year of study on average ambient temperature and sum of precipitation during the breeding season (May-June). ***Significant values P< 0.05 are in bold.

| **Sources of variation** | **Estimate** | **SE** | **t** | **P** |
| --- | --- | --- | --- | --- |
| *Temperature* |  |  |  |  |
| **Intercept** | **-94.61** | **26.13** | **-3.62** | **<0.001** |
| **Year of study** | **0.05** | **0.013** | **4.07** | **<0.001** |
|  |  |  |  |  |
| *Sum of precipitation* |  |  |  |  |
| Intercept | 13.00 | 11.33 | 1.15 | 0.258 |
| Year of study | -0.005 | 0.006 | -1.04 | 0.305 |

**
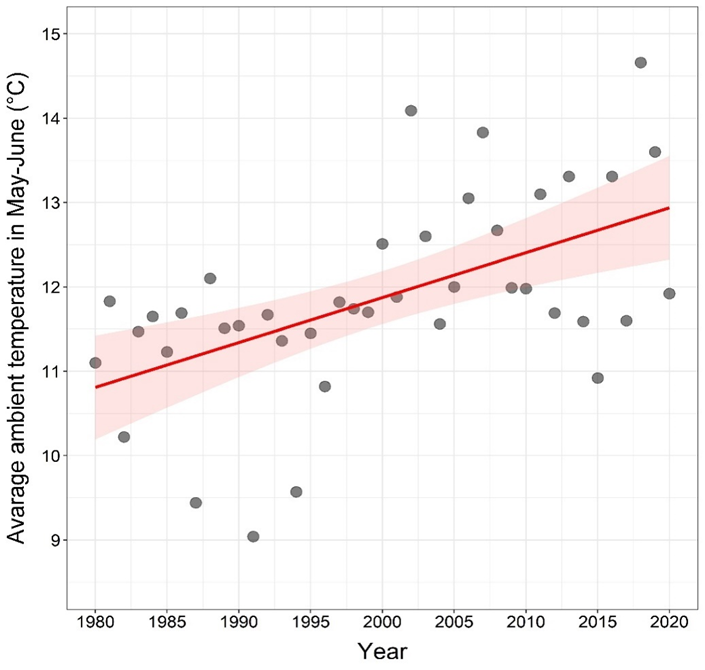
**

Figure SM1. Average ambient temperature during breeding season (May-June) on Gotland Island between 1980 and 2020.


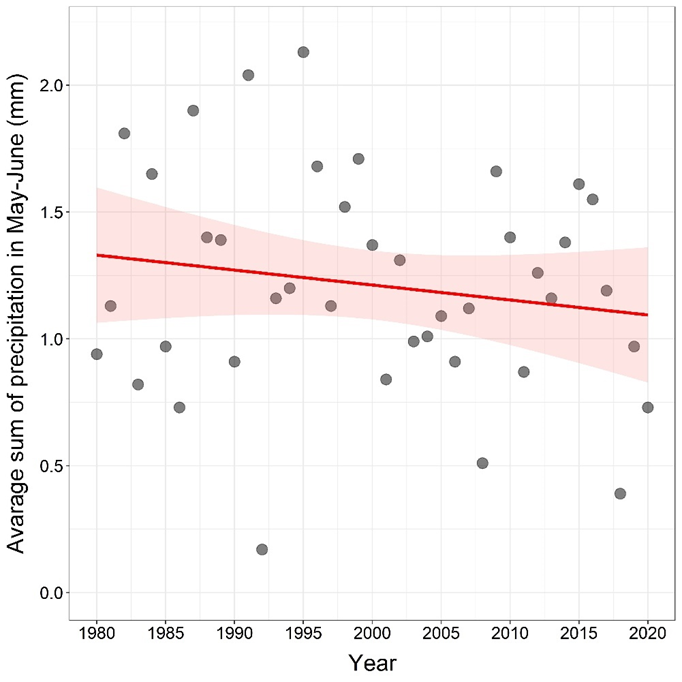


Figure SM2. Average sum of precipitation during breeding season (May -June) on Gotland Island between 1980 and 2020.

Table SM2. Variance inflation factor (VIF) and tolerance calculated for each term in all examined generalized mixed models (GLMMs).

|  | **GLMM for hatchling number** | | **GLMM for fledgling number:**  **conditional component** | | **GLMM for fledgling number:**  **zero-inflated component** | | **GLMM for recruit number** | |
| --- | --- | --- | --- | --- | --- | --- | --- | --- |
|  | **VIF** | **Tolerance** | **VIF** | **Tolerance** | **VIF** | **Tolerance** | **VIF** | **Tolerance** |
| **Female age** | 1.13 | 0.89 | 1.10 | 0.91 | - | - | 1.10 | 0.91 |
| **Female condition** | 1.08 | 0.93 | 1.06 | 0.94 | 1.05 | 0.95 | 1.06 | 0.94 |
| **Within-year temperature: incubation** | 1.44 | 0.70 | 1.92 | 0.52 | 1.81 | 0.55 | 1.76 | 0.57 |
| **Between-year temperature:**  **incubation** | 1.05 | 0.95 | 1.78 | 0.56 | 1.81 | 0.55 | 1.90 | 0.53 |
| **Within-year precipitation: incubation** | 1.10 | 0.91 | 1.25 | 0.80 | 1.26 | 0.79 | 1.31 | 0.76 |
| **Between-year precipitation: incubation** | 1.05 | 0.95 | 1.09 | 0.92 | 1.07 | 0.94 | 1.05 | 0.95 |
| **Within-year temperature: nestlings** | - | - | 2.40 | 0.42 | 1.94 | 0.51 | 2.57 | 0.39 |
| **Between-year temperature: nestlings** | - | - | 2.09 | 0.48 | 2.13 | 0.47 | 2.01 | 0.50 |
| **Within-year precipitation: nestlings** | - | - | 1.36 | 0.73 | 1.32 | 0.75 | 1.36 | 0.73 |
| **Between-year precipitation: nestlings** | - | - | 1.35 | 0.74 | 1.34 | 0.74 | 1.20 | 0.83 |
| **Laying**  **date** | 1.72 | 0.58 | 3.71 | 0.27 | 3.37 | 0.30 | 3.80 | 0.26 |
| **Clutch**  **size** | 1.18 | 0.85 | 1.17 | 0.86 | 1.28 | 0.78 | 1.07 | 0.87 |

Table SM3. The correlation matrix for continuous explanatory variables in a subset of data analyzing hatchling number (A), fledgling number (B), and recruit number (C). Pearson's correlation coefficients are shown.

A

|  | **Within-year**  **temperature:**  **incubation** | **Between-year**  **temperature:**  **incubation** | **Within-year**  **precipitation:**  **incubation** | **Between-year**  **precipitation:**  **incubation** | **Laying**  **date** | **Clutch**  **size** |
| --- | --- | --- | --- | --- | --- | --- |
| **Within-year**  **temperature**  **incubation** | 1.00 | - | - | - | - | - |
| **Between-year**  **temperature:**  **incubation** | 0.01 | 1.00 | - | - | - | - |
| **Within-year**  **precipitation:**  **incubation** | -0.06 | -0.02 | 1.00 | - | - | - |
| **Between-year**  **precipitation:**  **incubation** | 0.00 | -0.22 | 0.04 | 1.00 | - | - |
| **Laying**  **date** | 0.42 | -0.11 | 0.19 | 0.25 | 1.00 | - |
| **Clutch**  **size** | -0.11 | 0.02 | -0.04 | -0.08 | -0.40 | 1.00 |

B

|  | **Within-year**  **temperature:**  **incubation** | **Between-year**  **temperature:**  **incubation** | **Within-year**  **precipitation:**  **incubation** | **Between-year**  **precipitation:**  **incubation** | **Within-year**  **temperature:**  **nestlings** | **Between-year**  **temperature:**  **nestlings** | **Within-year**  **precipitation:**  **nestlings** | **Between-year**  **precipitation:**  **nestlings** | **Laying**  **date** | **Clutch**  **size** |
| --- | --- | --- | --- | --- | --- | --- | --- | --- | --- | --- |
| **Within-year**  **temperature:**  **incubation** | 1.00 | - | - | - | - | - | - | - | - | - |
| **Between-year**  **temperature:**  **incubation** | 0.01 | 1.00 | - | - | - | - | - | - | - | - |
| **Within-year**  **precipitation:**  **incubation** | -0.13 | -0.01 | 1.00 | - | - | - | - | - | - | - |
| **Between-year**  **precipitation:**  **incubation** | 0.02 | -0.18 | 0.06 | 1.00 | - | - | - | - | - | - |
| **Within-year**  **temperature:**  **nestlings** | -0.07 | 0.03 | 0.18 | -0.01 | 1.00 | - | - | - | - | - |
| **Between-year**  **temperature:**  **nestlings** | -0.02 | 0.58 | 0.02 | -0.06 | 0.02 | 1.00 | - | - | - | - |
| **Within-year**  **precipitation:**  **nestlings** | 0.22 | -0.01 | -0.26 | 0.03 | -0.07 | 0.00 | 1.00 | - | - | - |
| **Between-year**  **precipitation:**  **nestlings** | 0.02 | 0.09 | -0.04 | -0.15 | 0.01 | -0.48 | -0.01 | 1.00 | - | - |
| **Laying**  **date** | 0.38 | -0.09 | 0.13 | 0.30 | 0.41 | -0.03 | 0.22 | -0.06 | 1.00 | - |
| **Clutch**  **size** | -0.11 | 0.02 | -0.04 | -0.09 | -0.13 | 0.00 | -0.08 | 0.05 | -0.41 | 1.00 |

C

|  | **Within-year**  **temperature:**  **incubation** | **Between-year**  **temperature:**  **incubation** | **Within-year**  **precipitation:**  **incubation** | **Between-year**  **precipitation:**  **incubation** | **Within-year**  **temperature:**  **nestlings** | **Between-year**  **temperature:**  **nestlings** | **Within-year**  **precipitation:**  **nestlings** | **Between-year**  **precipitation:**  **nestlings** | **Laying**  **date** | **Clutch**  **size** |
| --- | --- | --- | --- | --- | --- | --- | --- | --- | --- | --- |
| **Within-year**  **temperature:**  **incubation** | 1.00 | - | - | - | - | - | - | - | - | - |
| **Between-year**  **temperature:**  **incubation** | 0.02 | 1.00 | - | - | - | - | - | - | - | - |
| **Within-year**  **precipitation:**  **incubation** | -0.08 | -0.02 | 1.00 | - | - | - | - | - | - | - |
| **Between-year**  **precipitation:**  **incubation** | 0.03 | -0.22 | 0.07 | 1.00 | - | - | - | - | - | - |
| **Within-year**  **temperature:**  **nestlings** | 0.06 | 0.08 | 0.22 | -0.04 | 1.00 | - | - | - | - | - |
| **Between-year**  **temperature:**  **nestlings** | -0.04 | 0.65 | 0.04 | -0.09 | 0.04 | 1.00 | - | - | - | - |
| **Within-year**  **precipitation:**  **nestlings** | 0.17 | -0.02 | -0.30 | 0.06 | -0.12 | 0.02 | 1.00 | - | - | - |
| **Between-year**  **precipitation:**  **nestlings** | 0.01 | 0.10 | -0.07 | -0.11 | 0.00 | -0.32 | -0.04 | 1.00 | - | - |
| **Laying**  **date** | 0.19 | 0.26 | 0.16 | 0.29 | 0.44 | 0.02 | 0.17 | -0.03 | 1.00 | - |
| **Clutch**  **size** | -0.05 | -0.08 | -0.04 | -0.10 | -0.13 | 0.02 | -0.05 | 0.04 | -0.38 | 1.00 |
